# The Influence of Local Habitat and Microclimate on the Levels of Secondary Metabolites in Slovak Bilberry (*Vaccinium myrtillus* L.) Fruits

**DOI:** 10.3390/plants9040436

**Published:** 2020-04-01

**Authors:** Zuzana Vaneková, Miroslav Vanek, Jaroslav Škvarenina, Milan Nagy

**Affiliations:** 1 Department of Pharmacognosy and Botany, Faculty of Pharmacy, Comenius University in Bratislava, 83232 Bratislava, Slovakia; nagy@fpharm.uniba.sk; 2 Department of Environmental Engineering, Faculty of Ecology and Environmental Sciences, Technical University in Zvolen, 96001 Zvolen, Slovakia; vanek@tuzvo.sk; 3 Department of Natural Environment, Faculty of Forestry, Technical University in Zvolen, 96001 Zvolen, Slovakia; skvarenina@tuzvo.sk

**Keywords:** bilberry, anthocyanins, flavonoids, tannins, environmental influence, soil

## Abstract

The berries of Vaccinium myrtillus L. are usually collected in the wild for the purpose of being a food source. They are naturally high in phenolic compounds, which possess antioxidative properties, so the berries are therefore often labeled as “functional foods”. This study evaluated seven samples of bilberry fruits from different locations in Slovakia for the content of the main phenolic compounds (anthocyanins, flavonoids and tannins) using European Pharmacopoeia 9 spectrophotometric methods. A thorough analysis of environmental factors showed that several phenolic constituents are closely corresponding with their respective environments, as well as with each other. The environmental factors with statistically significant correlations in this study are altitude, habitat type, sunlight exposure, and soil carbon content. Our findings suggest that the berries collected at sunny sites with no topsoil damage contain more phenolic compounds. The lowest amounts of phenolic compounds were found in samples from dense forests or with visible soil erosion and windthrow damage. The negative effect of windthrow damage on the levels of secondary metabolites in bilberry fruits has been described for the first time. This study observed no relationship between the amount of phenolic compounds and soil pH, soil nitrogen levels, or slope exposition.

## 1. Introduction

Bilberry (*Vaccinium myrtillus* L., Ericaceae) fruits have been an important part of local diets in many countries, including Slovakia. They are valued for their pleasant taste and aroma and are often processed into jams, preserves, juices, and alcoholic beverages. They are rich in anthocyanins which make for the intense dark purple coloration of the fruit, as well as all processed foods made from the berries. Their high market value is caused by their relatively difficult availability—bilberry bushes only grow in wild, montane areas. It is not possible to cultivate them due to very specific soil demands and the fruit harvesting is a tedious, tiring work, as it is done using either hands or small harvesting rakes [1,2]. 

Bilberries have also been used as a medicinal herb to treat various disorders. In the Slovak traditional medicine, bilberry leaves are used as a remedy against diarrhea, diabetes, urinary and gallbladder diseases, and rheumatism. Fruits are used in teas, juices, wine, tinctures, and capsules against fever, cold and night blindness and as adstringent tonic against diarrhea and stomach diseases [3]. European Medicines Agency considers bilberry fruits a traditional herbal medicinal product that is used either for treatment of mild diarrhoea and oral mucosa inflammation [4], or for treatment of capillary fragility and heavy legs caused by venous circulatory disturbances [5]. 

The above-mentioned relative unavailability and the impossibility of controlled cultivation make the quality control even more important. Especially for the medical purposes, bilberry fruits need to fulfill the pharmacopoeia quality standards: a minimum 1.0% of tannins, expressed as pyrogallol, in dried fruits [6] and a minimum 0.3% of anthocyanins, expressed as cyanidin 3-*O*-glucoside chloride, in fresh fruits [7]. There have been numerous studies in different countries showing that the levels of secondary metabolites may vary wildly depending on the harvest [8,9,10,11,12,13]. The results seem conflicting at best—the only proved and explained correlation is between the amount of sunlight and the amount of secondary metabolites, mainly anthocyanins and flavonoids: plants growing in sunny habitats produce fruits richer in these important antioxidant compounds [10,13,14].

There have been several studies that observed fruit yield and total biomass of bilberry plants in relation to soil parameters, in particular mineral nutrition and phosphorus [1]. Fertilization with nitrogen has been shown to increase the fruit mass and number of seeds in bilberries [15]. Forestry is another factor in bilberry undergrowth coverage; it has been shown that clear-cutting, stump harvesting, mire draining, and liming reduces the bilberry abundance, mainly due to the changes and disturbances of soil, as well as the loss of microclimate and humidity [16]. To the best of our knowledge, there has not yet been a study which would compare the soil quality parameters to the levels of secondary metabolites in bilberry fruits. 

There has been a previous study of bilberries in Slovakia [17] but the authors have made no attempt to evaluate the environmental influence on the amounts of the secondary metabolites. 

The aim of this work was to assess the amounts of selected secondary metabolites (total phenolics, anthocyanins, flavonoids and tannins) in seven samples of Slovak bilberry fruits collected at seven different sites and analyze the impact of growing conditions. In order to investigate the environmental influence, this research aimed to collect as much environmental data about the harvest sites as possible. This includes the site characteristics (latitude, longitude, altitude, slope orientation, and vegetation cover), the weather characteristics (cloudiness on the day of the harvest) and the soil characteristics (soil type, pH, levels of organic C and N).

## 2. Results 

### 2.1. Environmental Evaluation of Sample Locations

**Figure 1:**
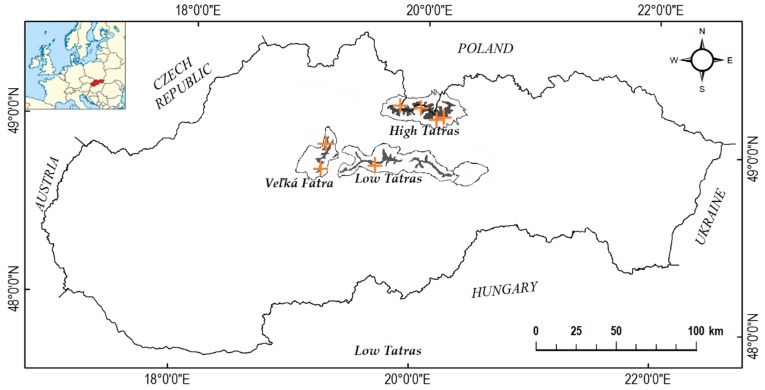
Locations of bilberry collection sites (marked in yellow crosses) in three mountain ranges in Slovakia.

**Figure 2:**
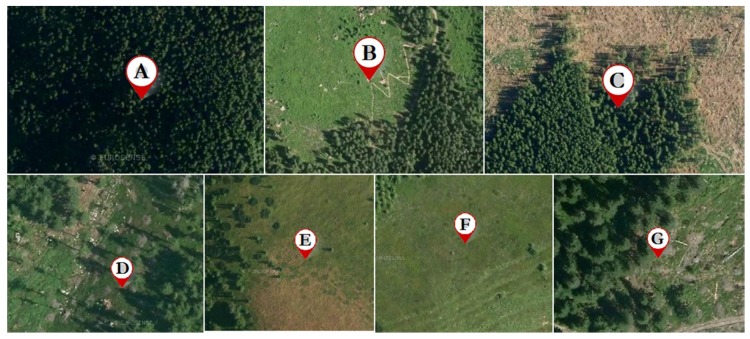
Satellite images of the harvest sites [18]. Satellite imagery is from 2015 (a year before the sample harvest).

The environmental values for all sample locations are listed in Table 1 and the sample locations are visualized in Figure 1 and Figure 2.

It is apparent that the samples come from wide range of habitats (from dense forest to open plain) in Table 1 and wide range of weathers on the day of collection (from completely overcast to sunny) in Table 2. There is an apparent difference between the soil samples in the levels of organic carbon: the two highest levels come from locations with notable windthrow damage, which points towards an increased decomposition of forest debris. 

### 2.2. Content of Phenolic Compounds

**Figure 3:**
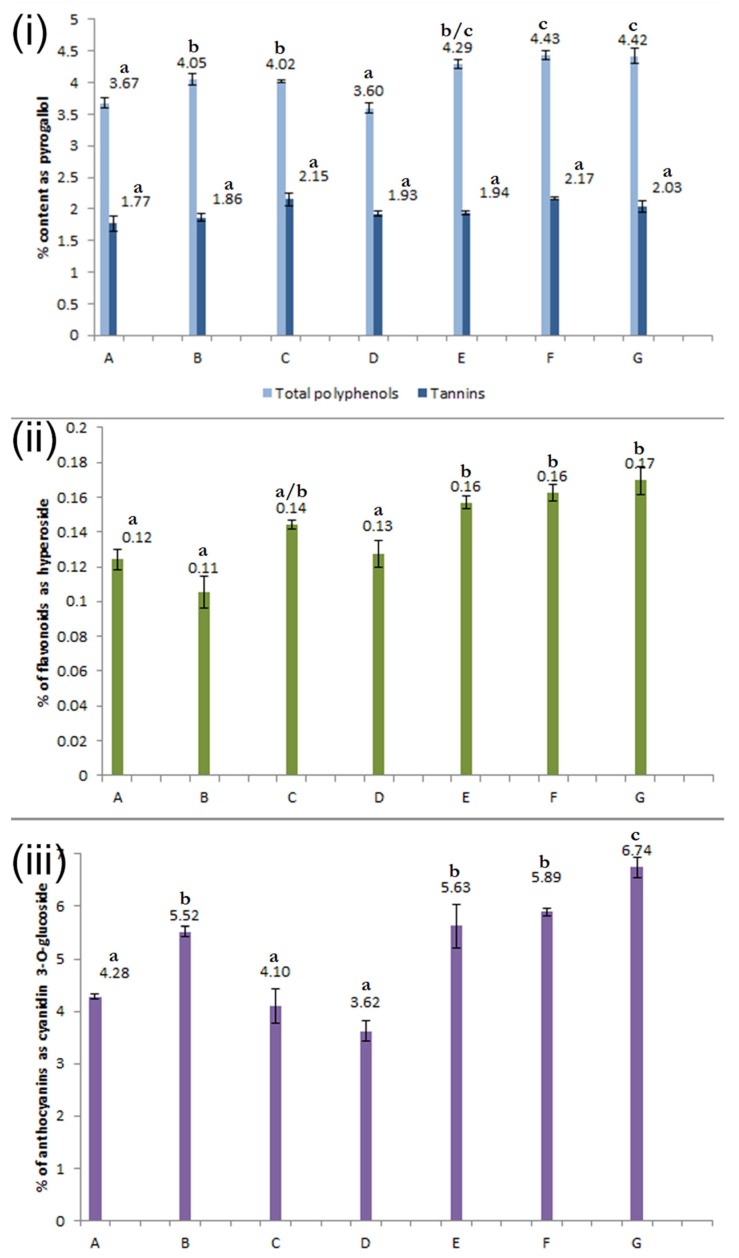
Contents of the phenolic compounds in the bilberry samples: total polyphenols and tannins (i), flavonoids (ii) and anthocyanins (iii). Content is expressed as % of the reference compound of sample dry weight (DW). Different letters (a–c) denote statistically significant differences among growing locations at *p* < 0.05 (*n* = 3).

The contents of the phenolic compounds in the bilberry samples can be seen in Figure 3, expressed as % of the reference compound of dry weight (DW). 

From Table 3 we can see that anthocyanins and flavonoids show the most significant correlation with the total phenolic content of bilberry fruits, therefore the level of total phenolics depends mainly on these compounds. The only metabolite pair that is not significantly dependent on each other are anthocyanins and tannins. 

### 2.3. Analysis of the Environmental Influence

To determine whether or not there is a relationship between environmental factors and the content of secondary metabolites, we performed a correlation analysis, the results of which can be seen in the Table 4. 

The factors with statistically significant correlations in this study are altitude, habitat type, sunlight exposure and soil carbon content. To an extent, these factors and their influence partially overlap. We will try to elucidate these relationships below in more detail. 

## 3. Discussion

### 3.1. Content of the Phenolic Compounds

There are significant differences in the levels of secondary metabolites between the samples from different locations. As can be seen in Figure 3, the samples can be grouped according to the statistically significant differences, where the members of each group show non-significant differences between each other but differ significantly from the rest of the samples. The only compound where this method was not applicable is tannins, where there were only a few instances of significant difference: samples A vs. C, A vs. F, and B vs. F, respectively. 

There can be no doubt that the levels of total phenolics in the samples of bilberry fruits are dependent on the levels of particular phenolic groups, namely anthocyanins, which make up the majority of the total phenolics. Phenolics in general, and flavonoids and anthocyanins in particular, are synthesized by related enzymatic pathways and are up-regulated by environmental factors, such as exposure to harsh weather conditions or increased UV irradiation [14]. Therefore, it is expected from a bilberry sample with low levels of total phenolics to have lower levels of flavonoids, anthocyanins and tannins as well [13]. 

These results are in accordance with the available literature. Secondary metabolites vary to some extent from country to country, as demonstrated on total anthocyanins in Table 5. 

These results are highly dependent on the methods used for extract preparation (ultrasonic bath, smaller particle size, and multiple extractions usually increase the extraction yield), methods for quantification (HPLC gives more detailed results than spectrophotometry), as well as on the mathematical model (e.g., numbers will vary depending on the standard compound used to express the results). 

### 3.2. Analysis of the Environmental Influence

Many scientific works have described multiple factors which can influence the amount of secondary metabolites in bilberry fruits. Among the most important are sunshine and UV irradiation exposure [10,14], altitude [13], latitude, climate, general state of weather throughout the year [10], and nutrient availability [15]. 

The previous studies found that tannins (which are one of the most important constituents of bilberry fruits) serve as a defense mechanism against pathogens, herbivores, and other pests. Their levels also seem to be closely related to the soil nutrient levels (plants produce more condensed tannins when growing on poorer soils), nutrient cycling in the soil and mycorrhizae fungi. They are only minorly influenced by UV irradiation [14,23,24]. 

The relationship between soil nutrients and condensed tannins here is reflected in the strong correlation between the amounts of soil carbon and the levels of anthocyanins. Bilberry fruits growing on poorer soils produce larger amounts of anthocyanins which are the precursors of condensed tannins (procyanidins, prodelphinidins, and their dimers and trimers) [25,26]. The berry samples from poorer soils produce larger amounts of anthocyanins, however the effect is not translated into a direct correlation between soil carbon and tannins, therefore there are most likely other, more powerful, environmental factors at work (see below). The same might be applied to the apparent relationship between tannins and altitude, since there is not any obvious or previously described mechanism that would explain such correlation. The one possible explanation might be the character of mycorrhizal fungi in different altitudes, which has been shown to have an impact on the tannin levels [24], and this might be a subject for further investigation. 

When it comes to habitat, we can divide the sample habitats into four groups:

- Dense forest (A, C)
- Windthrow glades (D, partially C)
- Harvest clearings (B, G)
- Naturally occurring grassy plains (E, F)

We can see that the amounts of secondary metabolites are significantly different between these four groups, which is also reflected by a significant correlation between the habitat factor and the level of total phenolics. On one hand, the tree coverage naturally lowers the amount of light that is able to reach the plants and stimulate the overproduction of flavonoids and anthocyanins [10,14]; this effect is clearly reflected in the significant positive correlation between the exposure factor and the amount of total anthocyanins. The exposure of the plants should always factor in the overgrowth coverage, since, as we can see in Table 4, neither the weather on the day of harvest, nor the weather conditions the week before the harvest show any significant correlation. 

On the other hand, we can observe the difference between the plants hit by a natural disaster (the massive windthrow that hit High Tatras in November 2004 and the following bark beetle outbreak and soil erosion), the plants affected by wood harvesting industry and the plants unaffected by drastic changes to their environment. While industrial harvesting and windthrow both dramatically reduce the tree coverage exposing the plants to an increased amount of sunlight, the windthrow causes more damage, disturbing the soil layer by the uprooted trees and the following soil erosion [27]. During industrial clear-cutting, it is possible to prevent leaching and surface erosion of nutrients through the presence of vegetation that retains nutrients in the ecosystem and the clearing (after an initial short period of biomass decrease and adjustment of plants) is beneficial for bilberry bushes [1]. 

On the first glance, it might seem paradoxical that the samples from the windthrow locations show the largest percentages of organic carbon in the soil, while simultaneously showing the lowest amounts of secondary metabolites. It might be easily understood by observing the character of vegetation and topsoil of the locations C and D, as can be seen on satellite images in Figure 2; the windthrow was followed by a decomposition of the destroyed vegetation, which increased the carbon content in the soil, but a simultaneous topsoil erosion had an overall stronger negative effect on the berry quality. The clearings caused by lumber harvest (B and G) did not suffer from such strong topsoil erosion and the undergrowth was kept largely intact.

High amounts of all compounds were observed in samples E and F, which were collected on wide grassy plains, and samples B and G, collected on forest clearings created by wood harvest. This is an important finding when it comes to berry harvest in Europe (perhaps only excluding Scandinavian countries), as the bilberry harvest is largely uncontrolled, performed by locals who treat it as a chance for seasonal income [1]. To ensure that the berries are the highest possible quality (for food or pharmaceutical industry), the obvious recommendation would be to preferably collect them at open locations where plants get enough sunlight all year round, and in areas with no topsoil damage. 

## 4. Conclusions

The chemical analysis confirmed that bilberry fruits are a rich source of dietary polyphenols, mainly anthocyanins and flavonoids, which often gets them labeled as “functional foods”. Our findings suggest that the berries collected at sunny sites with no topsoil damage contain more phenolic compounds. These findings are in accordance with the available literature. On the contrary, the lowest amounts of phenolic compounds were found in samples from dense forests or sites with visible soil erosion and windthrow damage to the flora. The negative effect of windthrow damage on the levels of secondary metabolites in bilberry fruits has been described for the first time. This study observed no relationship between the amount of phenolic compounds and soil pH, soil nitrogen levels, or slope exposition. These findings might help assure the higher quality of the berries harvested in their native habitats.

## 5. Materials and Methods 

### 5.1. Sample Collection

Fully ripe bilberry (*V. myrtillus* L.) fruits were selectively hand-picked during August and September 2016 in seven different locations of Slovak mountains (Figure 1, Table 1), along with soil samples. Only fully ripe fruits were picked; overripe and underdeveloped fruits were excluded. The locations were chosen as typical for bilberry, as *V. myrtillus* in Slovakia naturally occurs mainly in mountainous spruce stands, subalpine meadows and stands of dwarf pine (*Pinus mugo* Turra) [28]. Fruits were immediately dried at 40 °C (drying and heating chamber Binder ED 115, Binder GmbH, Germany), then ground into a fine powder using mortar and pestle cooled by liquid nitrogen (Messer Group GmbH, Germany). Resulting powder was sieved to achieve maximum particle size of 400 μm. Samples were frozen and stored at -10 °C in nitrogen atmosphere until analysis.

Soil samples were allowed to dry at the room temperature and stored in the dark until analysis.

### 5.2. Environmental Parameters

All assessed parameters are in Table 1. Latitude, longitude, altitude, exposition, habitat, and weather were evaluated on the day of the harvest. Detailed weather data were obtained from Slovak Hydrometeorological Institute. 

Soil type was obtained from Soil Map of Slovakia [29]. Soil pH and levels of organic carbon and nitrogen were obtained from Slovak National Forestry Center’s Central Forestry Laboratory. Soil samples were evaluated using accredited methods ISO 10390 (potentiometric measurement of pH in water suspension), DIN ISO 13878 and STN ISO 10694 (elemental analysis using a thermal conductivity detector to measure the amounts of carbon and nitrogen). The protocol can be accessed in Figure S1. 

From Table 1 we extrapolated several environmental factors with their respective values, which were used for statistical analysis:

- All (except one) sample locations are located on podzol-type soil; however, the parameters from soil quality analysis show large differences in soil nutrient levels—the poorest soils being samples A, E, and G, and the most nutritious being sample D.
- Exposition was expressed in degrees of a half-circle, with 0° being North and 180° being South (maximal value), since the sunlight availability is the lowest on northern slopes and highest on southern slopes.
- In order to assess the influence of the overgrowth coverage, a habitat factor was assigned, ranging from 1 (dense forest) to 10 (open grassy plain).
- According to the information from Slovak Hydrometeorological Institute, we selected the data about cloudiness (in %) on the day of sample collection, as well as the data about cloudiness from the week before each respective harvest date.
- Since the previous two factors have an additive effect on the final amount of sunlight that was able to reach the plants on the day of collection, we tentatively created a sunlight exposure factor, which was calculated by multiplying the % of clear sky by the habitat factor.

Latitude was excluded from this research, as there was not enough variation in the values to allow a conclusive analysis. 

### 5.3. Chemicals and Reagents

Acetic acid (concentrated 96%), Acetone p.a., Aluminum chloride, Ethyl acetate p.a., Folin–Ciocalteu reagent (phosphomolybdic-phosphotungstic acid), Hide powder CRS, Hydrochloric acid (concentrated 36%), Methanol p.a., Methenamine (hexamethylenetetraamine), Sodium carbonate and Sodium sulphate (anhydrous) were purchased from Centralchem, Slovakia. Distilled decarbonized water was used for all experiments.

Standards cyanidin 3-*O*-glucoside chloride, hyperoside and pyrogallol were purchased from Sigma – Aldrich, MO, USA. 

### 5.4. Determination of Phenolic Compounds

The methods used for the quantitative determination of selected phenolic compounds were derived from European Pharmacopoeia 9.0 (Ph.Eur.9), see below. All spectrophotometric measurements were performed on Genesys 6 spectrophotometer (Thermo Fisher Scientific, MA, USA). 

#### 5.4.1. Loss on Drying

The method according to Ph.Eur. 9 was used [30]. The results can be accessed in Table S1.

#### 5.4.2. Colorimetric Determination of Total Phenolics and Tannins

The method according to Ph.Eur. 9 [6], with slight modifications, was used. All the extraction and dilution operations were carried out in three parallel measurements and protected from light. In short, the accurately weighed powdered drug was extracted in boiling water for 30 min and diluted. 

Total phenolics: The primary extract was mixed with Folin–Ciocalteu reagent and diluted with a 290 g/L solution of sodium carbonate. After 30 min the absorbance was measured at 760 nm using water as the compensation liquid. 

Phenolics not adsorbed by hide powder: To 10.0 mL of the native extract, 0.10 g of hide powder was added and shaken vigorously for 60 min on a shaker. The mixture was then filtered, the filtrate was mixed with Folin–Ciocalteu reagent and diluted with a 290 g/L solution of sodium carbonate. After 30 min the absorbance was measured at 760 nm using water as the compensation liquid. 

The percentage content of tannins was calculated from the difference in the absorbances between total phenolics and phenolics not absorbed by hide powder. The content of total phenolics and tannins was expressed as % of pyrogallol equivalent and corrected for the loss on drying. 

#### 5.4.3. Colorimetric Determination of Total Flavonoids 

The method according to Ph.Eur. 9 [31] with slight modifications was used in three parallel measurements. In short, the accurately weighted powdered drug was extracted twice with acetone with a hydrolyzing agent (hexamethylenetetramine and hydrochloric acid) by boiling under a reflux condenser for 30 minutes. Flavonoid fraction was extracted to ethyl acetate by partitioning. The solution for colorimetry was prepared by mixing the ethyl acetate fraction with aluminium chloride reagent and 5% V/V solution of concentrated acetic acid in methanol. 

The absorbance of the solution was measured after 30 min, by comparison with the compensation liquid (mixture of water and reagents) at 425 nm. The content of flavonoids was expressed as % of the hyperoside equivalent and was corrected for the loss on drying. 

#### 5.4.4. Colorimetric Determination of Total Anthocyanins

The method according to Ph.Eur. 9 [7] with slight modifications was used in three parallel measurements. In short, the accurately weighed drug was extracted in methanol for 30 minutes on a shaker for 30 minutes. The extract was diluted, mixed with 0.1% V/V solution of hydrochloric acid in methanol and used for colorimetry. 

The absorbance of the solution was measured at 528 nm, using a 0.1% V/V solution of hydrochloric acid in methanol as the compensation liquid. The content of anthocyanins was expressed as % of cyanidin 3-*O*-glucoside chloride equivalent and was corrected for the loss on drying. 

### 5.5. Statistical Analysis

The data were analyzed using the Microsoft Excel software. Data from the chemical analyses were tested for any differences among growing locations using one-way variance analysis (ANOVA) with post-hoc Tukey HSD test. The correlations between the values of the environmental parameters and the amounts of secondary metabolites was evaluated using Spearman’s Rho calculation. P-values of less than 0.05 were considered statistically significant. 

## Figures and Tables

**Table 1:** Environmental parameters of bilberry samples.

Sample	Location	Date of Collection	Altitude (AMSL)	Latitude/Longitude	Slope Exposition	Habitat (Habitat Factor)	Soil Type	Soil pH	Corg (%)	N (%)
A	Tichá dolina, High Tatras	27.9.2016	1370	49.18307000 19.92212	SW	Dense spruce forest (1)	Podzol	3.89	6.59	0.378
B	Zadná Látaná dolina, Western Tatras	14.9.2016	1380	49.22836667 19.75435	NW	Harvest glade in spruce forest (7)	Podzol	4.28	7.48	0.448
C	Pod Ostrvou, High Tatras	18.9.2016	1475	49.13546000 20.08881	S	Spruce forest near a windthrow glade (2)	Podzol	4.07	9.84	0.401
D	Nad Novou Poliankou, High Tatras	17.9.2016	1415	49.13455000 20.14306	S	Windthrow glade in spruce forest (7)	Podzol	4.01	14.9	0.698
E	Ráztocká Hoľa, Low Tatras	20.8.2016	1435	48.87607000 19.3856	S	Grassy plain (10)	Podzol	3.81	6.18	0.439
F	Krížna, Veľká Fatra	18.9.2016	1459	48.87528630 19.08415318	NE	Grassy plain (10)	Cambisol	4.09	7.06	0.505
G	Smrekovica, Veľká Fatra	8.9.2016	1380	49.00015167 19.20923333	SW	Edge of a harvest glade (8)	Podzol	3.93	5.84	0.406

**Table 2:** Weather conditions during the week before sample harvest in the respective harvest locations [Average day cloudiness in %, blue cells signify precipitation].

Sample	D–7	D–6	D–5	D–4	D-3	D–2	D–1	D
A	70	80	47	50	63	53	43	50
B	37	10	23	23	30	17	33	25
C	30	17	33	43	30	47	77	60
D	0	10	10	17	17	33	37	95
E	53	80	47	83	83	67	63	60
F	7	13	17	27	23	57	100	80
G	23	70	40	63	100	77	40	10

D = day of harvest; D–1 = one day before harvest; etc.

**Table 3:** Correlations between secondary metabolite contents, expressed as Spearman’s Rho and their statistical significance (*n* = 7).

	Total Flavonoids	Total Anthocyanins	Total Tannins
Total phenolics	0.714	0.929 **	0.643
Total tannins	0.786 *	0.393	
Total anthocyanins	0.679		

* - *p* < 0.05; ** - *p* < 0.01.

**Table 4:** Correlations between secondary metabolite contents and environmental factors expressed as Spearman’s Rho and their statistical significance (*n* = 7).

	Altitude	Slope Exposition	Habitat Factor	Weather at Harvest (Cloudiness)	Weather Previous Week (Cloudiness)	Exposure	Soil pH	C_org_ (%)	N (%)
Total polyphenols [%]	0.204	−0.514	0.820 *	−0.317	0.286	0.750	0.143	−0.607	0.107
Anthocyanins [%]	−0.136	−0.513	0.742	−0.563	0.393	0.821 *	−0.071	−0.821 *	−0.036
Flavonoids [%]	0.374	0.086	0.742	−0.035	0.143	0.393	−0.250	−0.536	0.036
Tannins [%]	0.782 *	0.001	0.585	0.317	0.001	0.214	0.250	−0.036	0.179

* - *p* < 0.05.

**Table 5:** Comparison of total anthocyanins content in bilberry fruits from available studies.

Country	Anthocyanin Content [mg/g]	Fresh Weight (FW) or Dry Weight (DW)	Source
Finland	12	FW	[19]
	5.2	FW	[9]
Sweden	15–39	DW	[10]
Denmark	17	DW	[10]
Poland	9.9	FW	[20]
Austria	17–20	DW	[8]
Italy	36.6	DW	[21]
Slovenia	3.7	FW	[22]
	1–3.3	FW	[13]
	12	FW	[11]
Montenegro	1.7	FW	[12]

## Supplementary Materials

The following are available online at https://www.mdpi.com/2223-7747/9/4/436/s1, Figure S1: Protocol from soil analysis from Slovak National Forestry Center’s Central Forestry Laboratory, Table S1: Loss on drying of bilberry fruit samples.
