# Supplementary material for: The Influence of Local Habitat and Microclimate on the Levels of Secondary Metabolites in Slovak Bilberry (Vaccinium myrtillus L.) Fruits
Source: Plants (Basel). 2020 Apr 1;9(4):436. doi: 10.3390/plants9040436 (PMC7238256; doi:10.3390/plants9040436)
Supplement: Supplementary file 1 [file plants-09-00436-s001.docx]

**Supplementary files**


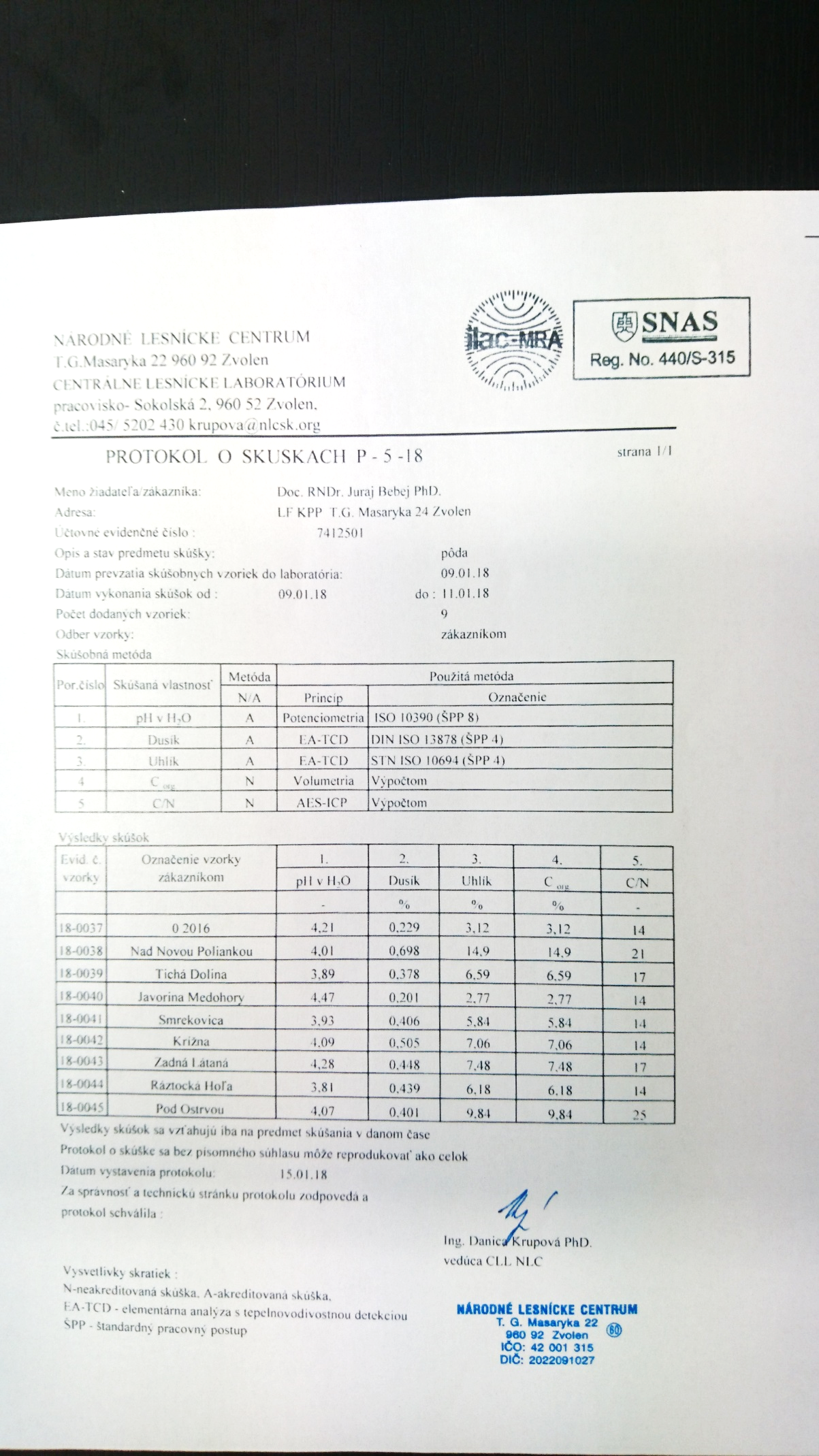


Figure S1: Protocol from soil analysis from Slovak National Forestry Center's Central Forestry Laboratory.

Table S1: Loss on drying of bilberry fruit samples.

| **Sample** | **Loss on drying (in % of weight)** |
| --- | --- |
| **A** | 9.347604 ± 0.166177 |
| **B** | 7.572594 ± 0.093043 |
| **C** | 7.189381 ± 0.053207 |
| **D** | 7.737792 ± 0.052487 |
| **E** | 11.07625 ± 0.074342 |
| **F** | 9.928549 ± 0.126976 |
| **G** | 9.354267 ± 0.14303 |
